# Exploring genome-transcriptome correlations in cancer

**DOI:** 10.1042/BST20240108

**Published:** 2025-02-05

**Authors:** Michael Ronemus, Daniel Bradford, Zachary Laster, Siran Li

**Affiliations:** Cold Spring Harbor Laboratory, Cold Spring Harbor, NY, U.S.A.

**Keywords:** genome-transcriptome relationship, dosage compensation, copy number variations, stromal mutations

## Abstract

We examine the complex relationship between genomic copy number variation (CNV) and gene expression, highlighting the relevance to cancer biology and other biological contexts. By tracing the history of genome-transcriptome correlations, we emphasize the complexity and challenges in understanding these interactions, particularly within the heterogeneous landscape of human cancers. Recent advances in computational algorithms and high-throughput single-cell multi-omic sequencing technologies are discussed, demonstrating their potential to refine our understanding of cancer biology and their limitations. The integration of genomic and transcriptomic analyses, which offers novel insights into tumor evolution and heterogeneity as well as therapeutic strategies, is presented as a crucial approach for advancing cancer research.

## Introduction

The exploration of genome-transcriptome correlations began outside of cancer biology in humans and model systems [1-3], with early studies exhibiting diverse mechanisms through which genomic alterations influence gene expression [4]. These studies found that copy number is not always directly scalable to gene expression levels. Kvitek et al. reported that only about 25% of genes with increased copy number in *Saccharomyces* strains showed elevated expression [5]. Further studies by Gasch and colleagues demonstrated that gene dosage compensation is frequently found in yeast [6,7], stabilizing gene expression levels against the effects of CNVs. In *Drosophila*, Sun et al. reported that triple X metafemales (XXX; AA) exhibit X-linked gene expression levels similar to those of double X normal diploid females (XX; AA), whereas autosomal gene expression in triple X metafemales is approximately one-third lower than that in double X females [8,9]. This is an inverse dosage effect where an increased number of X chromosomes leads to decreased autosomal expression. Dosage compensation within an aneuploid genome was also reported in mice [10]. In humans, Vilardell et al. [11] identified 324 genes across the genome with significant dosage effects in Down syndrome (trisomy 21), including 77 genes on chromosome 21 and 247 genes on other chromosomes. Further studies on Down syndrome show that epigenetic modifications, including H3K4me3 [12] and DNA methylation [13], are also associated with gene expression and domains of gene expression dysregulation. Considering these and many other studies, it is clear that various factors can affect genome-transcriptome correlations. CNV can include or exclude regulatory elements, which have varying effects on gene expression. In addition, epigenetic modifications, such as DNA methylation and histone modifications, can modulate gene expression independently of copy number. Other mechanisms, such as post-transcriptional processes, including RNA splicing [14-16], RNA editing [17-19], and RNA degradation [20-23], may also affect transcript and protein levels, adding another layer of complexity.

### Early genome-transcriptome correlational studies in cancer

In human cancers, the DNA sequence provides crucial phylogenetic information, while RNA profiles reveal cell types and states, enhancing our understanding of tumor heterogeneity and evolution. However, as is true in other biological contexts, the relationship between genomes and transcriptomes in cancer is not always straightforward.

Early studies using microarray assays on cell line models and bulk DNA from tissues suggested that relative gain or loss of a chromosome or chromosomal arm is usually coupled with a corresponding increase or decrease in the average expression level of all the genes within that region [24-26]. However, many studies have shown more complex relationships between genomic copy number and gene expression in human cancer, echoing the patterns between genome and transcriptome established in yeast, *Drosophila*, and mice, including the scaling of gene expression, dosage compensation, and effects on other chromosomes. First, an assessment of gene dosage effects showed that the relationship between DNA copy number changes and gene expression in tumors is not always proportional [27,28]. FitzPatrick et al. [29] found that the average level of transcription of a gene on a trisomic chromosome was increased only 1.1-fold compared with normal cells, demonstrating significant dosage compensation. Second, copy number changes on one chromosome sometimes lead to changes in gene expression levels on other chromosomes [29,30], indicating that local copy number changes in tumors can have global effects throughout the cell.

### Single-cell transcriptomics and copy number inference

The rapid growth of single-cell RNA sequencing technology (scRNA-seq) and the capture of more RNA transcripts from individual cells have led to the development of numerous algorithms to identify CNVs. from single-cell gene expression data. These include inferCNV [31,32] and HoneyBADGER [33], which are preferable for high-coverage assays like Smart-Seq, and copyKat [34], which is more applicable for high-throughput low-coverage scRNA-seq data. These algorithms have been widely applied in cancer research, and their usage has been facilitated in part by the adoption of high-throughput scRNA-seq assays [35,36], including several commercial platforms. The combination of these experimental and informatic techniques provides researchers with accessible insights into the genomic structure of tumor tissue. However, these algorithms tend to assume a positive correlation between genomic copy number and gene expression, which does not always hold due to the complex mechanisms influencing genome-transcriptome interactions previously mentioned.

Additionally, due to the inherent noise, sparsity, and complexity of single-cell RNA-seq data, these bioinformatics technologies generally specify the estimated genomic resolution of the inferred copy number events. These resolutions vary between samples as consequences of sequencing depth and cell number, requiring users to understand the algorithms, carefully examine the results, and validate the findings using other approaches, such as high-resolution single-cell DNA sequencing methods. Moreover, the efficacy of these algorithms is contingent on the accurate identification of normal cells [37], whose presence and characteristics can vary widely in tumor samples. Therefore, a direct measurement of both genomics and transcriptomics from a single cell is desirable for accurate comparison of the genome and transcriptome, especially in cancer cases where CNVs are frequent and genome-transcriptome relationships are particularly complex.

### DNA-RNA co-assays in single-cell cancer analyses

For single-cell technology, 2015 was a banner year, witnessing not only the incorporation of high-throughput microfluidic droplet platforms in single-cell RNA sequencing [35,36] but also the emergence of DNA-RNA co-assay techniques to study both the genome and transcriptome from single cells [38-40]. Although initially low-throughput, genome-transcriptome assays have expanded to include more cells [41-43] and more omics [44,45], such as methylation and chromatin structural information in addition to genome and transcriptome. These DNA-RNA co-assays have been used to study cancer cells with associated CNV information since they were first developed [38,39]. Despite being individual cell-based assays, the increase in cell numbers has made transcriptomic clustering possible [44,46,47]. This has allowed for the differentiation of cell types, particularly tumor cells and normal cells, using RNA clusters and CNV patterns. In addition to characterizing tumor heterogeneity in greater detail than single-omic single-cell technologies, Tang and colleagues [48] used a plate-based method combining RNA-seq and DNA sequencing (using a method called Multiple Annealing and Looping-Based Amplification Cycles (MALBAC) on tumor samples) to find that CNVs occur frequently in stromal cell types in cancer patients, including fibroblasts, endothelial cells, and immune cells. Specifically, they found that in the tumor microenvironment of human colorectal cancer, chromosome 7 amplification was common among cancer patients, with 26.4% (88 out of 333 cells from all patients) of fibroblast cells exhibiting this feature. They also found that sex chromosome loss was prevalent among both cancer patients and a non-cancerous control group; such genomic variations have also been reported frequently in elderly individuals lacking cancer diagnoses [49-58].

**Figure 1:**
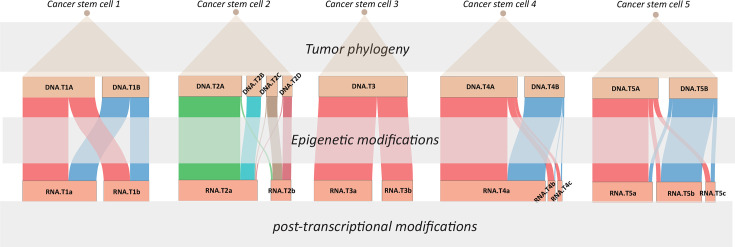
Genome-transcriptome relationships in five uterine cancer cases. This figure shows the diverse projection patterns between DNA subclones and RNA expression clusters across five uterine cancer cases, as reported in our recent study [61]. Each case illustrates how genomic variations (top blocks) correspond to transcriptomic profiles (bottom blocks), with lines connecting DNA subclones (e.g. DNA.T1A, DNA.T2B) to their associated RNA clusters (e.g. RNA.T1a, RNA.T2b). In addition to the explicit connections between genome clones and transcriptional clusters obtained directly from the data, the figure also presents the underlying temporal structure, including ‘tumor phylogeny,’ ‘epigenetic modifications,’ and ‘post-transcriptional modifications,’ exhibiting the multi-layered regulatory mechanisms that govern the relationship between genomic variations and gene expression in cancer.

**Figure 2:**
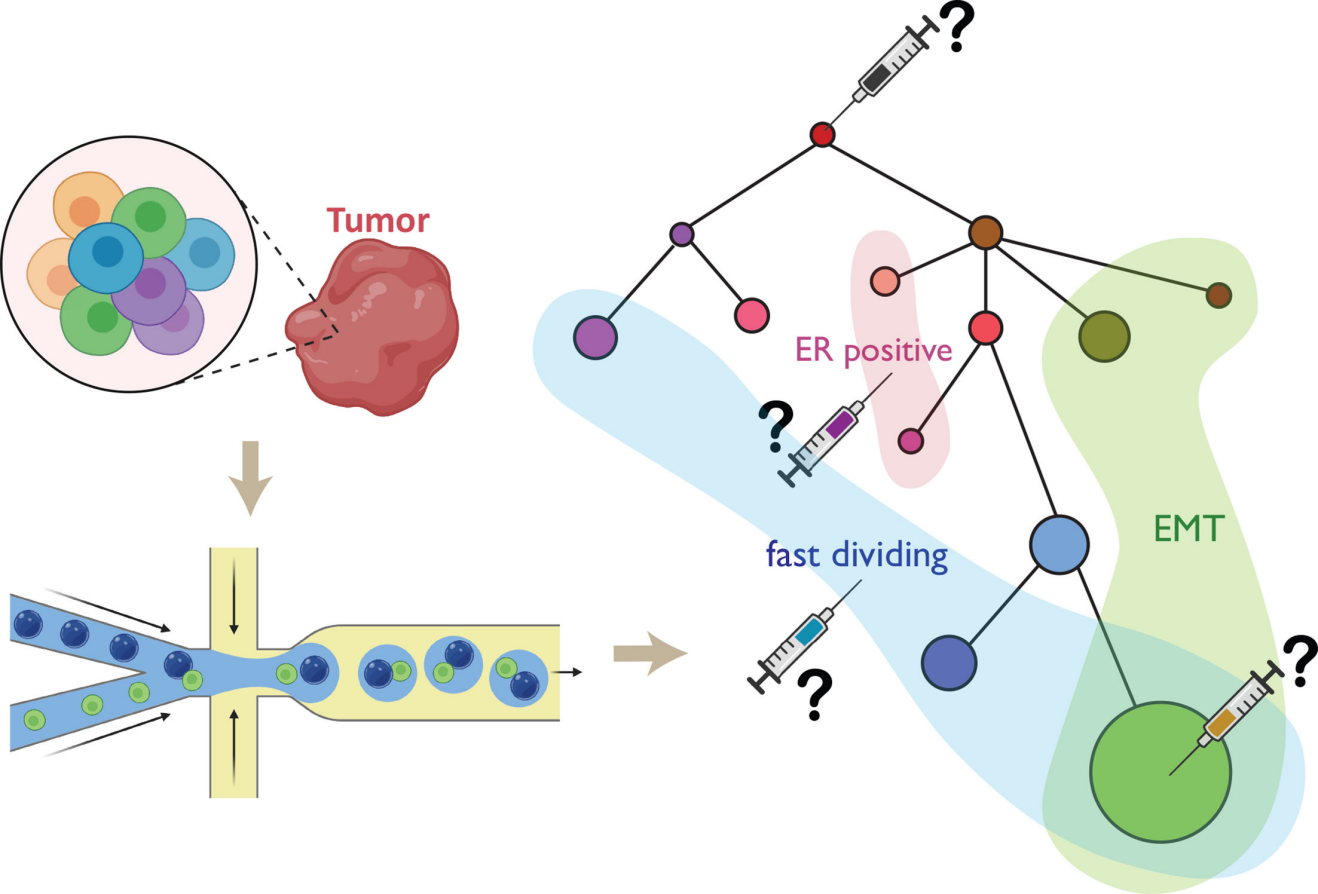
Tumor evolution and gene expression co-analyses highlight challenges in cancer drug design. High-throughput single-cell DNA-RNA co-analyses enable the simultaneous profiling of genomic and transcriptomic information from individual tumor cells. DNA data can be used to construct a phylogenetic tree, where each circle represents a distinct clone, and the size of the circle corresponds to the relative abundance of that clone. While individual DNA clones may exhibit unique gene expression profiles, some clones share common expression features linked to critical pathways, such as estrogen receptor (ER) signaling, epithelial-to-mesenchymal transition (EMT), rapid cell division, and others. These analyses pose important questions about designing effective cancer therapies and determining which subpopulations within the tumor should be prioritized as therapeutic targets.

In more recent years, high-throughput DNA-RNA co-assays using split-pool technology [59], droplet-based technology [60], or a combination of both [61] (developed by our team) have enabled researchers to study a larger number of cells in a more labor- and cost-effective manner. This boost in cell number enhances clustering accuracy and resolution, facilitating the detection of minor subpopulations that would be difficult to identify using earlier assays. The same population of cells can be clustered by RNA expression and genomic bin counts, respectively. The intuitive assumption has been that genomic and transcriptomic groupings would correspond directly, reflecting a one-to-one relationship. However, our results demonstrate that this is not always the case, as we have observed heterogeneity within and across genomic and transcriptomic layers. Relationships between tumor genome clones and tumor RNA clusters can vary significantly (Figure 1). The five cases we reported each demonstrated different tumor genome-transcriptome projection patterns, covering nearly all possible scenarios [61]. In the first case, we found that two tumor subclones both projected to two tumor RNA clusters and back-propagation of cluster identity from one omic to the other did not reveal any distinct pattern, implying that epigenetic modifications may have played a driving role in cancer development rather than genomic aberrations. In addition, we observed that some stromal mutations, particularly the loss of the X chromosome, were prevalent across patients and cell types. About one-third of each patient’s tumor-infiltrated plasma cells had lost one copy of the X chromosome, and these cells were clonal as determined by genome phasing and B-cell repertoire sequencing. These cells were also spatially localized, potentially indicating an area of unique immune resistance against tumor development. These observations echo previous bulk studies on cancer genomic CNV and gene expression in cancer while providing more detail, again revealing interesting, though often not straightforward, interactions between cancer genome and transcriptome. These results also revealed that whereas differences in genomic variations between two groups of tumor cells may lead to differences in the expression of some genes, this separation is not always the most biologically significant for the combined population. RNA clustering, or separation, might follow rules such as epithelial-to-mesenchymal transition (EMT) or estrogen receptor differences, which are unrelated to genomic clustering but biologically more significant. Although personalized drugs are typically designed to target RNA expression or antigen markers [62-65], greater consideration should be given to targeting root causes at the level of DNA clones (Figure 2).

### Future directions and clinical implications

With the aid of many new high-throughput assays, researchers have begun to observe correlations in more cases and across more cancer subtypes. We are gaining a better understanding of the correlation between cancer stage and patients’ clinical outcomes, which will undoubtedly require much larger patient cohorts. Although this review focuses primarily on the relationship between CNVs and gene expression, single-nucleotide variations [66,67] also exhibit complex relationships with cancer phenotypes and represent another interesting area of study. With higher yield, cleaner separation between different genomic layers, and potentially more information on DNA methylation and chromatin accessibility, we can map genome-transcriptomic correlations at much higher resolution, both at cluster and single-cell levels. This will help us more accurately identify tumor phylogeny, the founding clone, and the regulatory mechanisms driving gene expression changes within each cell. In addition to exploring the relationship between genome-transcriptome correlation and clinical outcomes, it would be interesting to combine these analyses with spatial sequencing technologies [68-70], which have progressed rapidly in recent years. This latter approach allows us to visualize the location of different genomic clones and expression clusters, showing where the mutant tumor and stromal cells reside and how these cells either assist or combat cancer expansion and metastasis. Integrating spatial information with genome-to-expression analyses could provide valuable insights into tumor biology and the tumor microenvironment, enabling the study of how hypoxic regions, immune suppression, and tumor boundaries influence the organization and interaction of different clusters and clones.

From a biological perspective, there is still much to uncover about which mutations drive cancer to exhibit various phenotypes, possess hallmarks of cancer [71], and become aggressive and metastatic. First, many primary tumors exhibit multiple subclones by the time cancer is initially detected and surgery is performed. If tumor phylogeny information can be obtained through genomic data such as CNVs, loss of heterozygosity, or SNVs, we could reconstruct the pseudotime development of cancer, identifying both the origin of cancer and its most recent clone. This information may offer valuable insights into which clone should be targeted for treatment, potentially guiding therapeutic strategies such as targeting the roots of the cancer clones with specific drugs. If the treatment does not target the original clone—the founding population that initiated the tumor—but instead focuses on the most abundant and prominent cell population identified by RNA or protein expression through RNA sequencing or histopathology, the cancer may recur. This recurrence happens because the original root clone still exists, even if it persists as a minor population before or after treatment, as these cells may not be affected by the drugs designed for other populations. Second, given the complex relationship between genomic clones and transcriptomic clusters [61], epigenetic modifications such as DNA methylation, chromatin accessibility, and chromosome conformation can modulate dosage compensation triggered by changes in copy number. Integrating epigenomic and transcriptomic multiomic technologies [41,72-78] with genomic data in a high-throughput manner will uncover more genome-level relationships between DNA and RNA. Third, although RNA expression data are highly informative about cell types and states and are often considered a key readout in phenotypic studies, RNA decay and post-transcriptional modifications can significantly influence protein expression and consequently the cell’s response to drugs. Technologies such as CITE-seq [79] have been applied in cancer and immunology [80,81] to further explore this relationship between RNA levels and protein expression. In addition, genome-to-expression analyses can also reveal adaptive mechanisms of therapy resistance, such as hypoxia-induced transcriptomic changes [82] linked to specific genomic alterations [83] or the emergence of therapy-resistant subclones exhibiting EMT signatures [84,85] or up-regulated immune checkpoint molecules [86]. These insights could facilitate the development of combination therapies targeting both the root clone and adaptive pathways. We are only beginning to understand the genome-transcriptome correlation in cancer and its clinical implications. With the continuous development of single-cell multiomic technologies and computational algorithms, along with the increase in sample sizes, our understanding of cancer biology will deepen, providing further clarification on the relationship between genome and transcriptome, proteome, phenotype and treatment options.

**Perspective**

- The integration of recently developed genomic and transcriptomic analyses provides novel insights into tumor evolution and heterogeneity and represents a crucial approach for advancing cancer research.
- Although initially believed to be positively correlated, the relationship between copy number and gene expression varies widely between tumors and is complex in nature.
- Integrating new sources of data, such as epigenomics, analysis of RNA dynamics, and spatial sequencing, can solidify the relationships between genome, transcriptome, and protein expression, which will allow for more targeted therapies.

## Open access statement

This article has been published open access under our Subscribe to Open programme, made possible through the support of our subscribing institutions, learn more here: https://portlandpress.com/pages/open_access_options_and_prices#conditional

## Abbreviations

CNV: copy number variation
EMT: epithelial-to-mesenchymal transition
ER: estrogen receptor
MALBAC: Multiple Annealing and Looping-Based Amplification Cycles
SNV: single-nucleotide variation
scRNA-seq: single-cell RNA sequencing technology
